# Massive upper tract hematuria following decompression of chronic PUJ obstruction: A rare but clinically significant complication

**DOI:** 10.1016/j.eucr.2025.103267

**Published:** 2025-11-12

**Authors:** Vismaya K.B., Haris Chirakkal, Ramkumar Aiyappan

**Affiliations:** a Department of Urology, GMC, Trivandrum, Kerala; b Department of Urology, GMC, Trivandrum, India; c Department of Urology, GMC, Trivandrum, India

## Abstract

Hematuria after urinary decompression is common in chronic lower urinary tract obstruction but rarely originates from the upper tract. We report a 58-year-old woman with long-standing PUJ obstruction who developed massive renal pelvic hemorrhage following DJ stent placement. She presented with gross hematuria, hemoglobin drop, hypertension, and imaging showing a large renal pelvic clot. Conservative management stabilized her, and delayed surgery corrected the obstruction. This case highlights upper tract decompression hematuria as a rare yet potentially life-threatening complication requiring prompt recognition and careful management.

## 1 Introduction

Hematuria is a relatively common complication following decompression of chronic urinary retention, particularly when involving lower urinary tract pathologies such as bladder outlet obstruction. The classical mechanism involves ischemia-reperfusion injury of the chronically overdistended bladder wall, resulting in mucosal friability and bleeding. Typically, this bleeding is mild and self-limiting. In stark contrast, hematuria originating from the upper urinary tract following the relief of chronic hydronephrosis is rarely encountered in clinical practice and remains sparsely documented in the literature.

Ureteropelvic junction (UPJ) obstruction is characterized by a blockage that hinders the flow of urine from the renal pelvis into the proximal ureter, resulting in progressive dilation of the collecting system and possible damage to the kidney. Only a limited number of cases describing spontaneous hemorrhage in patients with ureteropelvic junction obstruction have been documented in the literature.^1^ Ureteropelvic junction (UPJ) obstruction may arise from intrinsic factors—most notably, an overabundance of connective tissue and reduced smooth muscle content within the ureteral wall—or from extrinsic causes, such as aberrant renal vasculature, including accessory, early-branching, or lower pole vessels. However, the exact role of these anomalous vessels remains a subject of ongoing debate, as it is often unclear whether they are the primary cause of obstruction or merely coexist as anatomical variants alongside an underlying intrinsic defect.^2^ The paucity of such reports may stem from underrecognition, the subtlety of presentations, or an assumption of bladder etiology by default.

This case report aims to expand the differential diagnosis of post-decompression hematuria by presenting a unique case in which massive renal pelvic hemorrhage occurred after DJ stenting for Ureteropelvic junction obstruction. The patient required transfusion support and experienced significant hypertensive sequelae. A review of recent literature suggests that this

underappreciated complication may warrant greater clinical awareness, especially in patients with longstanding upper tract obstruction and comorbidities such as hypertension or renal insufficiency.

## 2 Case report

A 58-year-old female with a history of type 2 diabetes mellitus and longstanding hypertension presented with intermittent right-sided abdominal pain of one-year duration. Initial laboratory investigations revealed a serum creatinine level of 2.49 mg/dL, raising concerns for underlying obstructive uropathy.

**Fig. 1:**
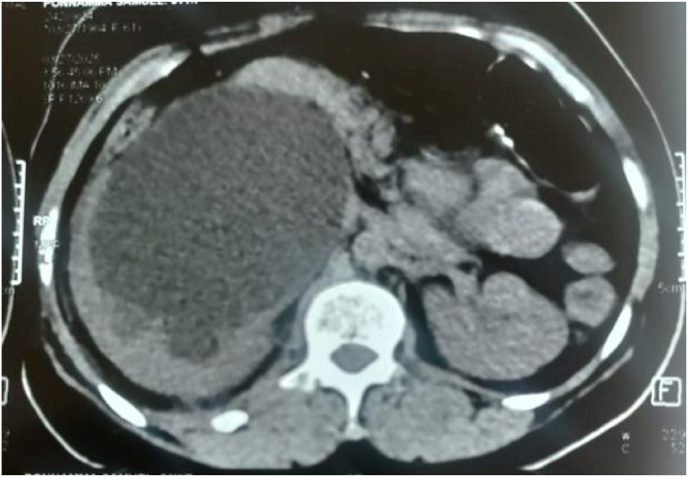
Pelvi-ureteric junction obstruction before relief.

Non-contrast computed tomography (NCCT) of the abdomen confirmed gross right hydronephrosis with significant cortical thinning (Fig. 1). The renal pelvis was markedly dilated, measuring up to 85 mm in diameter, with an abrupt narrowing observed at the uretero-pelvic junction, suggestive of high-grade obstruction. Given the degree of obstruction and impaired renal function, the patient underwent cystoscopy, right ureteroscopy (URS), and DJ stent placement. Intraoperative findings included a kink in the upper ureter just below the uretero-pelvic junction, raising suspicion of extrinsic compression. Post-procedure, her renal function improved significantly, with serum creatinine decreasing to 1.4 mg/dL.

**Fig. 2:**
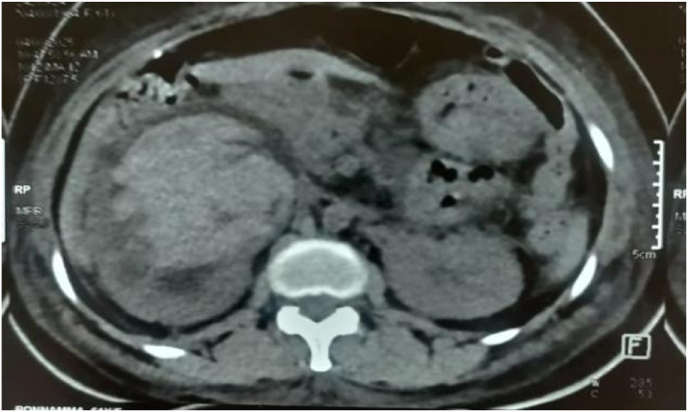
Pelvi ureteric junction bleed as evidenced by pelvic clot.

**Fig. 3:**
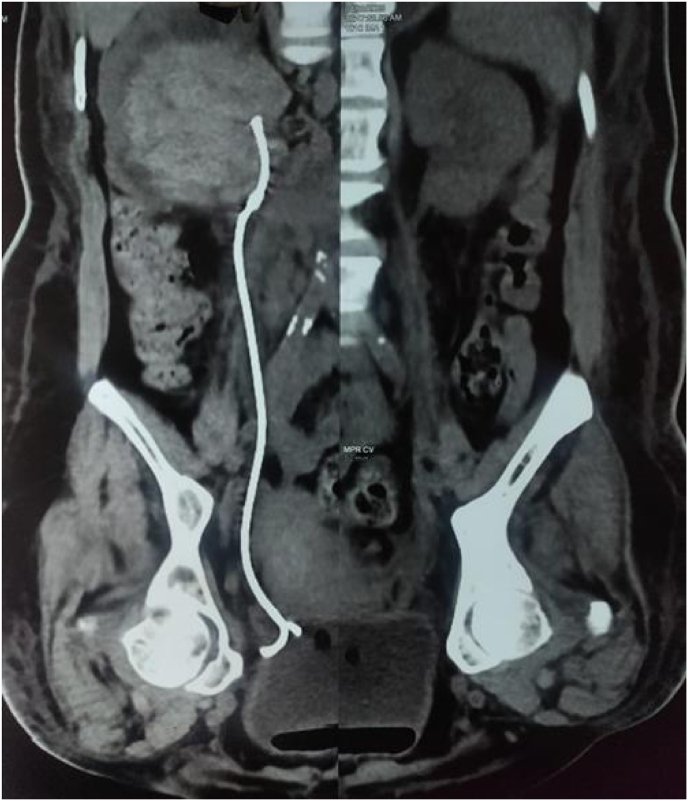
Gross hydronephrosis with pelvic clot post stenting with DJ stent in situ.

However, within 8 hours, the patient developed gross hematuria and a hypertensive crisis, with blood pressure peaking at 220/110 mmHg. A repeat CT scan revealed a large, nonenhancing hyperdense lesion within the renal pelvis measuring 7.3 × 5.7 cm, consistent with a blood clot(Fig. 2). Associated findings included ballooning of the calyces, thinning of the renal parenchyma, and moderate perinephric fat stranding. CT urography further confirmed a markedly dilated extrarenal pelvis (4.7 cm) with abrupt tapering at the Ureteropelvic junction. The previously placed DJ stent remained in situ(Fig. 3).

The patient required transfusion of three units of packed red cells. Despite the severity of the bleeding, she remained hemodynamically stable. Conservative management, including bed rest, fluid resuscitation, antihypertensive therapy, and close monitoring, resulted in gradual clinical improvement. She was discharged in a stable condition with instructions for followup. On her three weekly follow up, the patient's right DJ stent was removed. Post removal her serum creatinine levels remained stable at 1.2mg/dl (Table 1).

**Table 1:** Tabular column depicting variation in serum creatinine and hemoglobin pre and post procedure.

Postoperative Day	Hemoglobin (g/dL)	Serum Creatinine (mg/dL)
Preoperative	9.7	2.6
POD 1	8.2	2.5
POD 2	7.1	2.0
POD 3	6.5	1.8
POD 4	6.7∗	1.6
POD 5	7.9∗	1.4

**Fig. 4:**
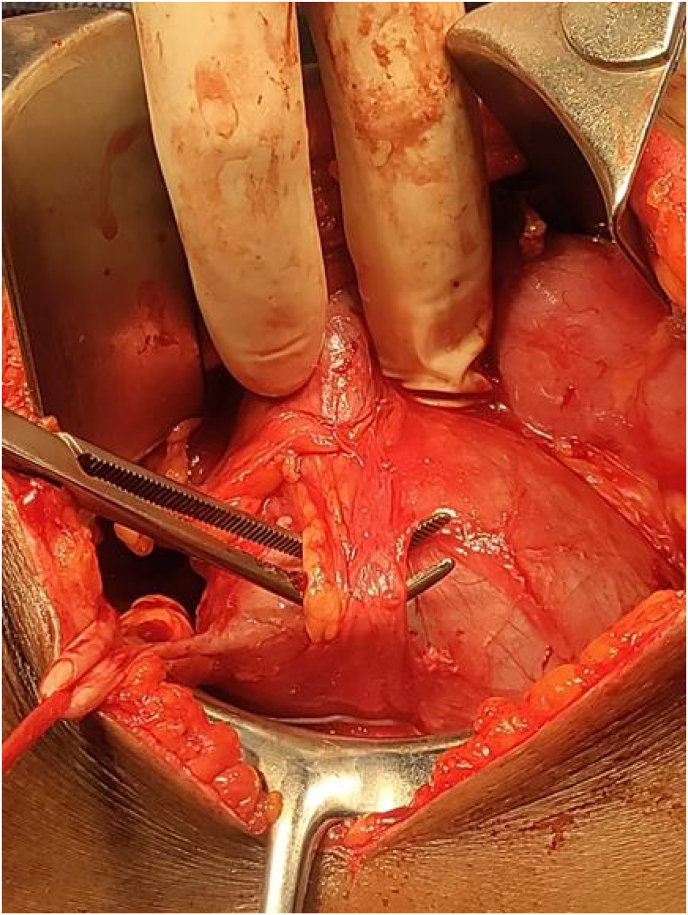
Intra operative photo showing anterior crossing vessels.

Two months later, the patient was readmitted for definitive surgical management of the underlying obstruction. Intraoperatively, an anterior crossing vessel compressing the uretero-pelvic junction (Fig. 4) was identified. Dismembered pyeloplasty was performed.

No clot was observed intraoperatively, suggesting that it likely underwent spontaneous lysis. The postoperative course was uneventful. The patient's blood pressure normalized and was subsequently controlled with intermittent doses of cilnidipine.

## 3 Discussion

Hematuria is defined as the abnormal presence of red blood cells in the urine. It is classified into microscopic hematuria, defined as the presence of three or more red blood cells per high-power field on urine microscopy, and gross hematuria, where blood is visibly present in the urine, causing discoloration ranging from pink to cola-colored urine 3 discussion.

Hematuria after decompression of chronic urinary retention is a well-known clinical entity. Hematuria has been described following bladder drainage in 2 %–16 % In most cases, it originates from the bladder and is attributed to reperfusion injury of ischemic bladder mucosa. However, bleeding from the upper urinary tract following decompression of longstanding hydronephrosis, although exceedingly rare, represents a distinct and potentially more severe complication.^4^.

The current case is notable for several reasons. First, the hemorrhage clearly originated from the renal pelvis rather than the bladder, as evidenced by imaging findings. Second, the magnitude of bleeding led to hemodynamic compromise, renovascular hypertension, and required blood transfusion—a combination rarely reported in the literature.

The pathophysiology of decompression-related upper tract hematuria is hypothesized to involve sudden changes in intrapelvic hydrostatic pressure. Chronic obstruction leads to dilation and congestion of the collecting system, including the subepithelial capillary networks. Rapid decompression may result in shearing or rupture of these vessels. Additionally, the sudden re-expansion of previously compressed parenchyma and collecting structures could contribute to mechanical trauma and vascular injury.

Recent reports have echoed similar themes. In 2021, Chung et al. described a case of perinephric hematoma following DJ stenting in a patient with obstructive

hydronephrosis^5^^.^ Persaud et al. documented spontaneous renal pelvic hemorrhage requiring nephrectomy in a patient with previously undiagnosed Ureteropelvic junction obstruction obstruction.^1^ Praveen et al., in 2018 reported isolated renal pelvis bleeding following decompression, highlighting the potential for severe upper tract hemorrhage even in the absence of trauma or infection.^6^

It is worth noting that although a crossing vessel was ultimately identified and addressed during surgery, the acute hemorrhagic event was not directly attributable to the vascular anomaly but rather to the physiological consequences of decompression. This distinction is crucial, as it shifts the focus from anatomic to hemodynamic considerations in the pathogenesis of upper tract bleeding.

The management of such cases remains nuanced. Conservative therapy is often appropriate in hemodynamically stable patients, with surgical intervention reserved for definitive correction of the underlying obstruction. In this patient, a staged approach allowed for stabilization of renal function and control of hypertension before addressing the anatomical etiology.

## 4 Conclusion

This case underscores the need to broaden the differential diagnosis of post decompression hematuria to include upper tract sources, particularly in patients with chronic ureteropelvic junction obstruction. The phenomenon of upper tract decompression hematuria, though rare, can lead to serious clinical consequences including massive hemorrhage, acute kidney injury, and renovascular hypertension. Early recognition and tailored management—combining conservative resuscitation with delayed surgical correction—are essential for optimal outcomes.

As urologists increasingly rely on minimally invasive interventions for upper tract obstruction, awareness of potential decompression-related complications must be heightened. Further research and case reporting are warranted to better characterize this uncommon but clinically significant entity.

## CRediT authorship contribution statement

**Vismaya K.B.:** Writing – original draft, Conceptualization. **Haris Chirakkal:** Writing – review & editing, Visualization, Supervision, Conceptualization. **Ramkumar Aiyappan:** Writing – review & editing.
